# Development and psychometric evaluation of a scale assessing nursing students’ knowledge, attitudes, and practices regarding evidence-based care bundles

**DOI:** 10.1186/s12912-026-04488-0

**Published:** 2026-02-28

**Authors:** Tuğba Demiroğlu Dost, Gülistan Yurdagül

**Affiliations:** 1 Vocational School of Health Services, University of Karamanoğlu Mehmetbey University, Karaman, Turkey; 2 Department of Nursing, Faculty of Health Sciences, Osmaniye Korkut Ata University, Osmaniye, Turkey

**Keywords:** Care bundle, Nursing, Evidence-based care, Scale development

## Abstract

**Background:**

Care bundles improve the quality of care through evidence-based practices; however, no standardized tool exists to assess nursing students’ competencies related to their use. This study aimed to develop a valid and reliable scale to address this gap.

**Materials and methods:**

This methodological study was conducted with 340 nursing students enrolled in the 3rd and 4th years between September and October 2024. The initial draft of the scale consisted of 17 items was evaluated by six experts to established content and face validity. Internal consistency was examined through a pilot study. Construct validity was assessed using exploratory factor analysis (EFA), and the confirmatory factor analysis (CFA) was performed to test the model fit of the factor structure obtained from EFA. Items 11 and 16, which negatively affected construct validity, were removed from the scale. Test-retest analysis was used to assess temporal stability, and Cronbach’s alpha coefficient was calculated to evaluate reliability.

**Results:**

The final version of the scale consisted of three subdimensions, namely f “knowledge”, “attitude” and “practice”. The KMO value of the scale was 0.88; Bartlett’s Sphericity Test Chi-Square value was χ2 = 1370.046; df = 136; *p*<0.001. The total variance explained by EFA was 50.39. The scale’s Cronbach’s alpha value was 0.82, and the sub-dimension values were calculated as knowledge 0.66, attitude 0.62 and practice 0.83.

**Conclusions:**

Based on the findings, the Care Bundle Knowledge, Attitude and Practice Scale meets content, construct and reliability criteria and can be considered a valid and reliable measurement tool for nursing students.

**Clinical trial number:**

Not applicable.

**Supplementary Information:**

The online version contains supplementary material available at 10.1186/s12912-026-04488-0.

## Background

Care bundles are systematic, evidence-based care approaches consisting of at least three practices designed to enhance the quality of patient care. First introduced in 2001 in the United States by the Institute for Healthcare Improvement [1, 2], they have since been implemented across a wide range of clinical settings, yielding consistently positive outcomes. Care bundles are tailored to specific patient groups, promote the standardization of care, and integrate interventions whose individual effectiveness has been established into a cohesive and holistic framework [3]. Through this approach, they improve patient safety, reduce hospital length of stay, increase patient satisfaction, and decrease healthcare-related costs [1].

The effectiveness of care bundles has been investigated in numerous clinical areas. Evidence indicates significant benefits in ventilator-associated pneumonia [4], central venous catheter–related infections [5, 6], neonatal and pediatric units [7, 8], adult intensive care units [4], pressure ulcers [9, 10], sepsis and delirium [11, 12], prevention of surgical site infections [13], fall reduction [14], and neonatal health practices [15]. Collectively, these findings provide a strong evidence base supporting the use of care bundles for both disease prevention and the improvement of care quality.

The effective implementation of care bundles requires adequate knowledge, appropriate resources, professional motivation, and interdisciplinary collaboration. As primary members of the multidisciplinary team and those directly responsible for patient care, nurses play a critical role in this process. Nurses’ knowledge, attitudes and practices related to care bundles are key determinants of successful implementation [1]. However, the existing literature reveals a notable lack of valid and reliable instruments designed to evaluate the knowledge, attitudes, and practice of both nurses and nursing students regarding care bundles.

Therefore, the present study aimed to develop a valid and reliable instrument to assess the knowledge, attitudes, and practices of nursing students with respect to care bundles. It is anticipated that this scale will serve as a valuable assesment tool in nursing education by enabling the evaluation of students’ competencies related to evidence-based care bundles. In addition, the instrument may be used in clinical settings to assess nurses’ proficiency. Ultimately, the scale is expected to contribute to improved care quality, enhanced patient safety, and the promotion of evidence-based nursing practices.

Although care bundles are widely implemented and have been shown to improve patient outcomes, there is currently no standardized, psychometrically validated instrument specifically designed to evaluate nursing students’ knowledge, attitudes, and practices related to care bundle implementation. This gap is particularly critical in the context of nursing education, as most existing research has focused primarily on clinical outcomes rather than educational competency assessment. Consequently, educators face challenges in systematically measuring students’ readiness for evidence-based care bundle implementation use and in identifying areas requiring curricular improvement.

This deficiency limits the ability of educators and academic institutions to determine learning needs, monitor the development of competencies, and promote the consistent and effective application of care bundles in clinical practice. Accordingly, the development of a valid, reliable and psychometrically robust instrument to evaluate nursing students’ knowledge, attitudes, and practices related to care bundle implementation is warranted. In response to this need, the present study sought to determine whether a valid and reliable scale could be developed to assess nursing students’ competencies in this domain.

## Materials and methods

### Aim of the study

The aim of this study was to develop a valid and reliable measurement tool to assess the knowledge, attitudes and practices of nursing students regarding care bundle implementation in patient care.

### Design of the study

This study was designed as a methodological research study.

### Setting of the study

The study was conducted in the department of nursing a state university between September and October 2024. The data collection process began with the pre-test application on September 23, 2024. The post-test application was conducted 4 weeks later, on October 14, 2024.

### Population and sample of the study

The study population consisted of students enrolled in the nursing department of a state university. The scale was initially developed with 18 items. Based on expert recommendations and because the content validity ratio of one item was below 0.42, and one item was revised, resulting in a 17-item draft scale. Based on the information that the sample size should be at least 5–10 times the number of items included in a scale [16], the study was conducted with 170 nursing students, using a pre-test and post-test (test–retest) design at a four-week interval, without sample selection.

### Inclusion criteria for the study

Participants were required to meet the following criteria:

- Being a registered and active undergraduate nursing student at the state university where the study was conducted,
- Continuing formal education during the study period,
- Participating in both the pre-test and post-test applications,
- Providing written informed consent,
- Being actively angaged in hospital clinical practice.

### Exclusion criteria for the study

Participants were excluded if they:

- Were absent from school during the data collection period due to reasons such as health problems, family loss, economic dificulties, or withdrawal from education,
- Participated simultaneously in another similar study, to prevent potential cross-interference.

### Data collection tools

#### Student identification information form

This form was developed by the researchers by reviewing the literature. It includes items related to age, gender, marital status, education level, academic year, and whether the care bundles was implemented in the clinical settings where students received practical training [17–21].

#### Care bundle scale development form

The care bundle scale was initially developed as an item pool consisting of 18 items, based on an extensive review of the literature [1–4, 6–8, 10, 11, 13, 18, 22]. Each item was rated on a 5-point Likert-type scale, ranging from “strongly disagree (1)” to “strongly agree (5)”. The total score obtainable from the scale ranged from 18 to 90, with higher scores indicating higher levels of knowledge, attitudes, and practices related to care bundle implementation.

To evaluate the content validity of the draft scale, opinions were obtained from a panel of six experts, including two experts in Turkish Language, two in Fundamentals of Nursing, and two in Internal Medicine and Public Health Nursing. Experts evaluated each item using the Lawshe method, classifying them as “(a) appropriate,” “(b) appropriate but requires revision,” or “(c) not appropriate” [23, 24]. In addition, the experts were provided written feedback and suggestions for each item.

The content validity ratio (CVR) for each item was calculated by dividing the number of experts who rated the item as “appropriate” by the total number of experts [25, 26]. According to Lawshe’s content validity criteria, the acceptable level of the content validity ratio (CVR) depends on the number of experts participating in the evaluation process. For assessments involving six experts, the critical CVR value is set at 0.42 based on Lawshe’s content validity table derived from a binomial probability model. In the present study, this criterion was applied, and items with CVR values below 0.42 were deemed insufficient and subsequently removed from the scale [23].

Based on expert evaluations and the calculated content validity ratio (CVR) values, one item that did not meet the minimum CVR threshold was removed from the scale. The remaining items were subsequently revised in accordance with qualitative feedback provided by the experts to enhance clarity, wording, and conceptual alignment, resulting in a 17-item draft version of the scale.

Beyond content validity, item comprehensibility was treated as a key element of face validity and was evaluated through expert assessment. Each item was reviewed in terms of clarity, ease of understanding, and the suitability of its wording. In line with the experts’ recommendations, minor revisions were made to several items to enhance their clarity and comprehensibility, thereby strengthening the face validity of the scale.

Following expert evaluation, the preliminary version of the scale was tested in a pilot study with 20 nursing students to **assess** item clarity and comprehensibility from the perspective of the target population. Participants provided feedback regarding item wording and ease of understanding. Based on this feedback, final revisions were made, and the scale was finalized prior to the main data collection (Additional file 1).

### Implementation of the study

After the pilot study, the finalized scale was administered to nursing students in classroom setting. The students were informed about the purpose of the study, and written informed consent was obtained. Students then completed the Student Identification Form and the Care Bundle Scale. Data were collected using a face-to-face survey method, and completion of the questionnaires required approximately 6–7 min.

To evaluate the temporal stability, a test–retest method was applied. The scale was administered at baseline and re-administered to the same participants four weeks later, with no intervention occurring between administrations. This procedure was used to determine whether the scale produced stable and consistent results over time.

### Data analysis

Data were analyzed using SPSS 24.0 and AMOS 21 software. Content validity was evaluated based on expert opinions. The adequacy of the sample size for factor analysis was assessed using the Kaiser–Meyer–Olkin (KMO) coefficient, and Bartlett’s Test of Sphericity was applied to examine item correlations.

Exploratory factor analysis (EFA) and confirmatory factor analysis (CFA) were conducted to assess the construct validity of the scale. Data from 170 participants ere used for EFA and data from 170 participants were analyzed for CFA. Factor loadings subdimensions and explained variance obtained from EFA were examined, model fit was tested using CFA [27]. In addition, relationships between item fit indices and subdimensions were evaluated. Cronbach’s alpha coefficient (α) was calculated to assess the internal consistency reliability of the scale. Because the pretest and post-test measurements were obtained from the same participants, the observations were not independent. Therefore, paired statistical techniques were employed, and test–retest reliability analysis was conducted to evaluate the temporal stability of the scale.

### Ethical aspect of the study

Ethical approval was obtained from the Kilis 7 Aralık University Non-Interventional Clinical Research Ethics Committee (Date: 27.03.2024 and Number: E-76062934-044-48650) and from the Karamanoğlu Mehmetbey University Faculty of Health Sciences, where the study was conducted. Participation was based on voluntariness, and participants were informed that their data would be handled in accordance with confidentiality principles, that they could withdraw from the study at any time, and that no financial compensation would be provided. Written and verbal informed consent was obtained from all participants. The study was conducted in accordance with the Declaration of Helsinki. The Guidelines for Reporting Reliability and Agreement Studies (GRRAS) were followed to ensure standardized reporting of reliability and agreement analyses of measurement instruments.

## Results

### Descriptive characteristics of student nurses

The age range of student nurses participating in this study ranged fom 19 to 25 years. Of the participiants, 57.1% were aged 19–21 years, 79.4% were female, 98.2% were single, 55.3% were 4th year students, while the remaining participiants were 3rd year students. Additionally, 98.2% of student nurses reported that they had not previously received training on care bundle practice, and 92.4% reported that care bundle practices were not implemented in the clinical settings where they complited their practical training.

The descriptive characteristics of the student nurses are presented in Table 1.

**Table 1:** Descriptive characteristics of the participants

	Group	f	%
Age	19–21 age	194	57.1
	22–25 age	146	42.9
Gender	Female	270	79.4
	Male	70	20.6
Marital Status	Married	6	1.8
	Single	334	98.2
Grade level	Level 3	152	44.7
	Level 4	188	55.3
Receiving Care Package Training	Yes	6	1.8
	No	334	98.2
Care Package Practice in the Clinic	Yes	26	7.6
	No	314	92.4

### Exploratory factor analysis (EFA)

To determine the suitability of the data for EFA, the Kaiser-Meyer-Olkin (KMO) coefficient and the Bartlett’s Test of Sphericity were examined (Table 2).

**Table 2:** Care bundle practice, knowledge and attitude scale KMO and bartlett sphericity test results

Kaiser-Mayer-Olkin (KMO) Value		0.88
Bartlett’s Sphericity Test	Ki Kare	1370.046
	Df	136
	Sig	0.00
Cronbach Alpha		0.82

For EFA to be conducted, the KMO value is required to be greater than 0.60 [27, 28]. In this study, The KMO value was calculated as 0.88, indicating excellent sampling adequacy. Bartlett’s Test of Sphericity was found to be statistically significant [χ2 = 1370.046; df = 136; *p*=0.000], demonstrating that the data were suitable for factor analysis.

The overall Cronbach’s alpha coefficient of the scale items was 0.82, indicating high internal consistency [28].

**Fig. 1:**
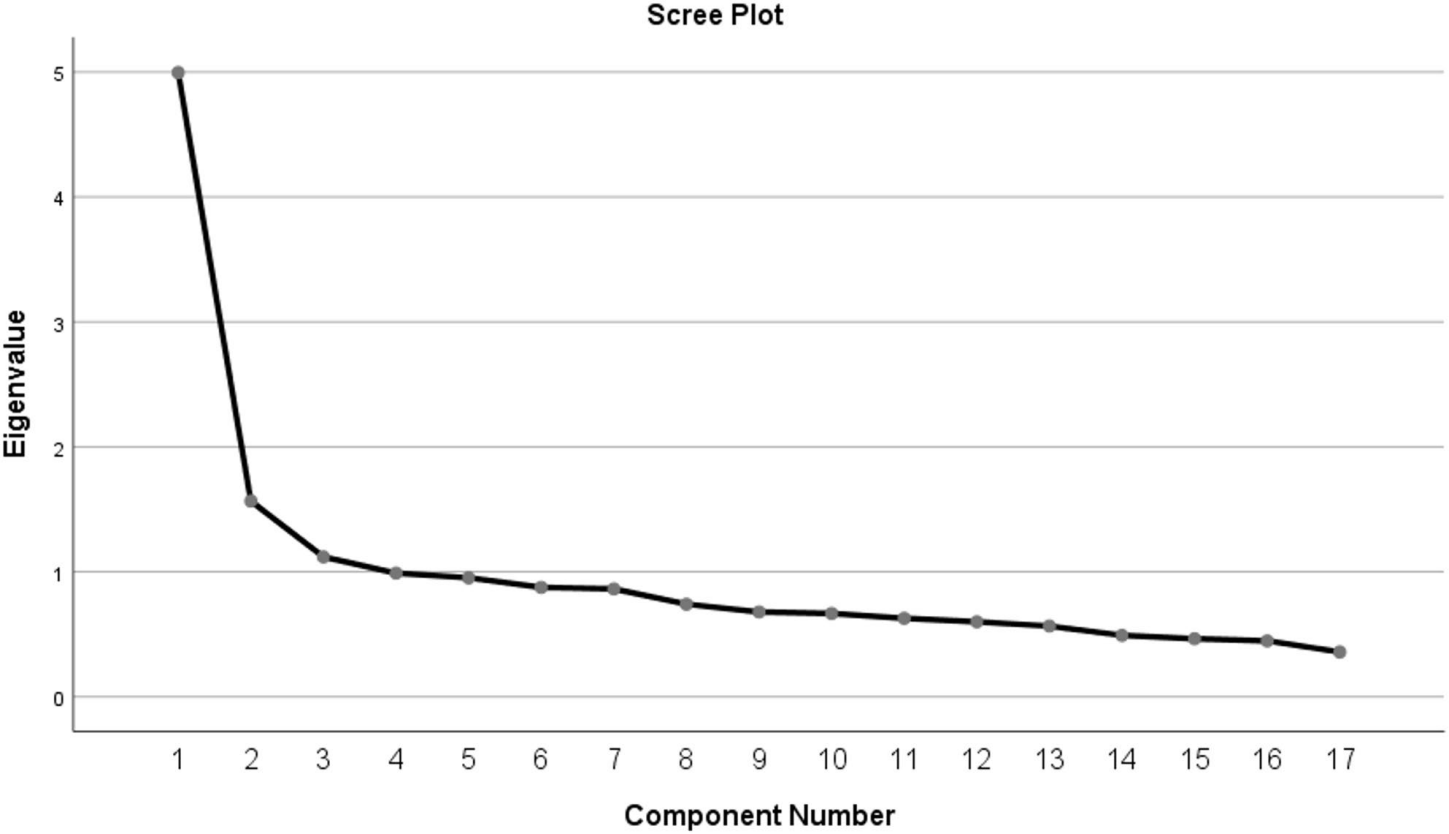
Scree plot graph for the eigenvalues of the factors

Acoording to the Kaiser-Guttman criterion, factors with eigenvalues greater than 1 were retained, and the scale was found to consist of three factors. The scree plot, illustrating the eigenvalues of the factors, is presented in Fig. 1.

In the literature, it is reported that a total variance explanation of 40% or higher is sufficient [29]. Additionally, factor loadings of at least 0.32, with cross-loadings below 0.30, are considered acceptable [27, 28]. Based on these criteria, two items (Items 11 and 16) with factor loadings below 0.40 were removed, and the analysis was repeated. No changes were observed in the factor structure or subdimensions following item removal. The final version of the scale consisted of 15 items, and the factor loadings and communalities of these items are presented in Table 3.

**Table 3:** Factor loadings and common factor variance

Sub-Dimensions	Item No	Items				Item Factor Loading
Practice	1	I know that the care package application aims to provide the highest level of care.				0.62
	2	I know that the care package is a set of methods used to standardize care and treatment in hospitals.				0.52
	3	I know that the care package is more of a customized care plan rather than a checklist.				0.55
	4	I can create the care package based on scientific evidence.				0.46
	6	I can prepare the care package according to the needs of the institution.				0.40
	8	I know that the care package should be implemented by a multidisciplinary team.				0.52
	13	I can implement the interventions in the care package simultaneously.				0.43
	14	I can implement all the interventions in the care package in order to be successful.				0.48
	17	I know that the care package application will increase the quality of health services.				0.56
Attitude	5	The interventions in the care package should be designed in a way that they can be applied to every patient.				0.45
	9	Care package applications should be used in a defined patient population in a specific place.				0.35
	12	The interventions in the care package should be applied in their entirety, paying attention to the all-or-nothing rule.				0.44
Knowledge	7	I know that the care package should be prepared specifically for the disease, symptom or medical condition, not for the patient.				0.40
	10	I know that care package applications include at least 3 and at most 5 applications that have been proven to be effective.				0.61
	15	I know that feedback should be given to practitioners in the care package application.				0.61
			Practice	Attitude	Knowledge	
Eigenvalue			4.84	1.18	1.03	
Explanatory Variance			34.57	8.45	7.37	
Explanatory Total Variance			**%50.39**			

As shown in Table 3, the total variance explained by the three- factor structure exceeded 50%, indicating a strong factor structure. Variance explanation rates between 40% and 60% are considered adequate in scale development studies [27, 30]. The factor loadings of the first subdimension ranged from 0.40 to 0.62 and included nine items. The second subdimension consisted of three items, with factor loadings ranging from 0.43 to 0.56, while the third subdimension also consisted of three items, with factor loadings between 0.40 and 0.61. Together, these three factors explained 50.39% of the total variance.

Based on expert consensus and the conceptual scope of the items, the factors were named accordingly. The first factor, explaining 34.57% of the total variance, was labeled “Practice”; the second factor, explaining 8.45%, was labeled “Attitude”; and the third factor, explaining 7.37%, was labeled “Knowledge”.

When Table 4 is examined, statistically significant correlations at the 0.01 level are observed among the scale’s subdimensions. The relationship between practice and attitude was moderate, between practice and knowledge was high, and between attitude and knowledge was **moderate**, with correlation coefficients ranging from 0.33 to 0.52. According to commonly accepted criteria, correlation coefficients of 0.10–0.30 indicate a small relationship, 0.30–0.50 a moderate relationship, 0.50–0.70 a high relationship, 0.70–0.90 a very high relationship, and 0.90–1.00 a perfect relationship [31].

**Table 4:** Correlation analysis results between factors

Factors	Practice	Attitude	Knowledge
Practice	1.00	0.49**	0.52**
Attitude		1.00	0.33**
Knowledge			1.00

**Significant correlative relationship at 0.01 level

### Item reliability analyses

To evaluate the extent to which the three factors measured the intended construct, item-total correlation coefficients were examined. All item–total correlation values were found to be 0.21 or higher, exceeding the recommended minimum value of 0.20 [29].

Additionally, an independent samples t-test was conducted to examine differences between the upper 27% and lower 27% groups based on factor scores.

As shown in Table 5, t-values ranged from 12.81 to 32.75, and the differences between the upper and lower groups were statistically significant at *p* < 0.05 for all factors. The mean scores of the upper 27% group were consistently higher than those of the lower 27% group. A similar pattern was observed for the overall scale scores.

**Table 5:** Discrimination of scale factor and total scores (t Test)

Sub-factors	Group	N	X	ss	r**	t test	
						t	p*
Practice	Subgroup %27	91	8.80	1.79	0.21	12.81	0.00
	Topgroup %27	91	11.67	1.61			
Attitude	Subgroup %27	91	8.21	1.60	0.22	13.16	0.00
	Topgroup %27	91	11.32	1.68			
Knowledge	Subgroup %27	91	26.70	4.97	0.38	22.03	0.00
	Topgroup %27	91	37.59	2.92			
Total	Subgroup %27	91	49.02	6.80	0.66	32.75	0.00
	Topgroup %27	91	66.64	3.65			

*p < 0.05; ** *r*: Factor total correlation

Following the removal of two items with low factor loadings, Cronbach’s alpha coefficients were recalculated. The reliability coefficient was 0.83 for the Practice subdimension, 0.62 for the Attitude subdimension, and 0.66 for the Knowledge subdimension. The overall internal consistency coefficient of the scale was 0.84. According to Sönmez and Alacapınar (2017) [32], internal consistency coefficients of 0.60 or higher are considered acceptable. Therefore, the reliability of the scale was deemed adequate.

### Confirmatory factor analyses (CFA)

**Fig. 2:**
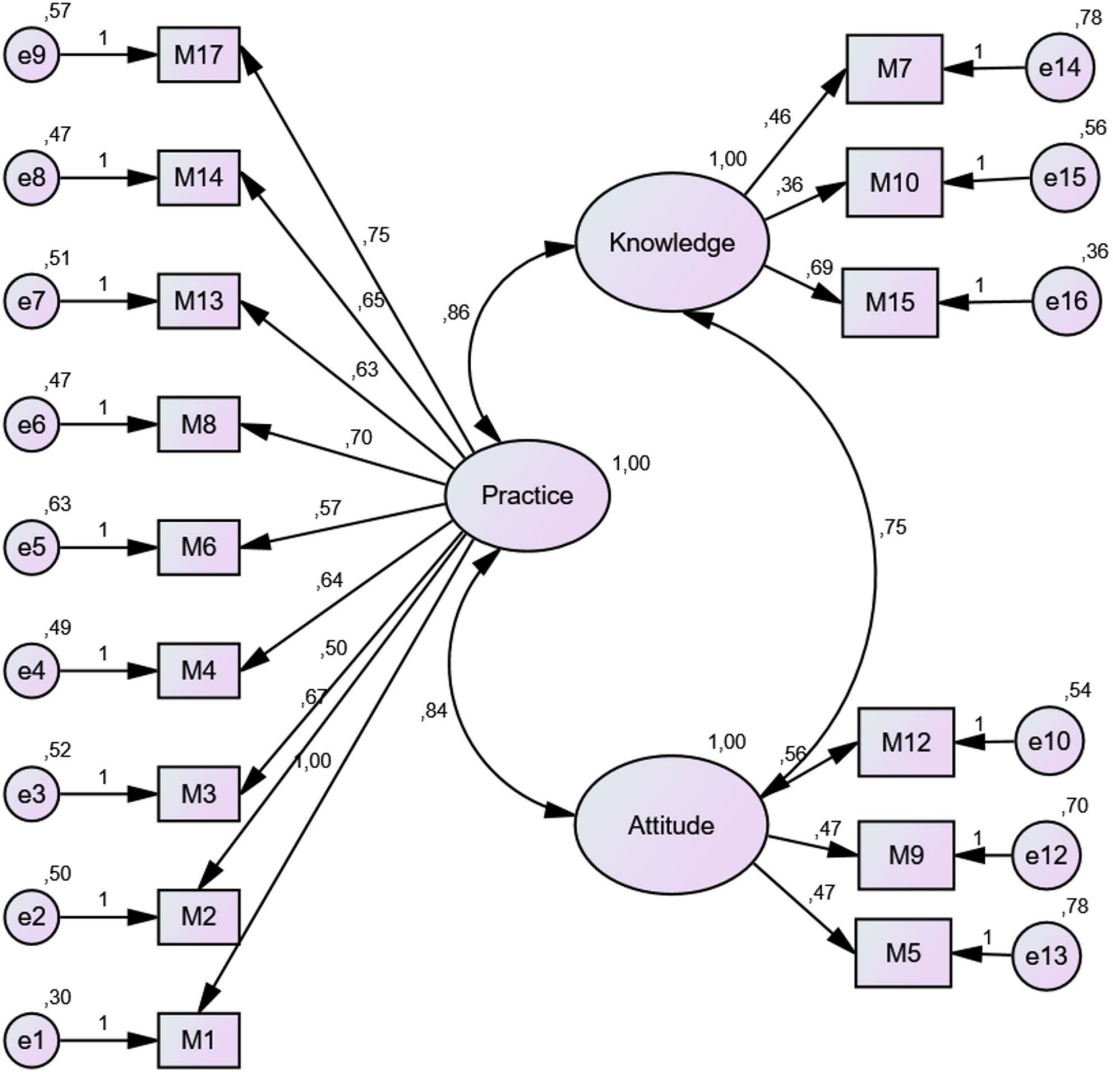
Sub-dimensions and standardized values of items of the care bundles application knowledge and attitude scale

To verify the factor structure identified through EFA, CFA was conducted. The CFA model consisted of 15 items and 3 sub-dimensions as seen in Fig. 2. The fit indices were evaluated without applying any model modifications.

The chi-square (X2) value of the model was 189.577 ± 85, with a X2/sd ratio of (2014.17/774) 2.23, indicating an acceptable model fit [33]. However, GFI was determined as 0.93, and AGFI as 0.90. Hooper et al. (2008) [34] stated that these values being ≥ 0.90 indicate a “good fit”. Of the fit indices, Normal Fit Index (NFI) is 0.90 and Non-Normal Fit Index (NNFI) is 0.91. Tabachnick and Fidell (2007) [35] reported that being ≥ 0.90 indicates a good fit. The Comparative Fit Index (CFI) value was determined as 0.91. It is reported that values of CFI indices above 0.90 are an indicator of good fit [28, 36]. It is seen that the standardized Root Mean Square Residual fit index (SRMR) is 0.14. Gökdemir and Yılmaz state that cases where the standardized RMR value is ≥ 0.08 are an indicator of good fit [36]. Another important index is Root Square Error of Approximation (RMSEA). The value was determined as 0.06. In the literature, an RMSEA value of ≤ 0.08 is accepted as an indicator of good fit (Gökdemir & Yılmaz, 2023 [36].

### Correlation between sub-dimensions

Within the CFA model, the standardized correlation coefficient between practice and knowledge was 0.86, between knowledge and attitude was 0.75, and between attitude and practice was 0.84. These correlation coefficients were positive and high, indicating strong relationships among the subdimensions. In linear relationships, correlation coefficients range from − 1 to + 1, where − 1 indicates a perfect negative relationship and + 1 indicates a perfect positive relationship [32].

### Test-retest reliability

To assess the temporal stability of the developed scale, a test-retest method was employed. The scale was re-administered to 170 participants four weeks after the initial application. The mean score of the first administration was 58.44 ± 7.81, while the mean score of the second administration was 57.74 ± 8.36.

Normality analysis indicated that skewness and kurtosis values were within the acceptable range of ± 1.5 (Skewness: −0.59 to − 1.12; Kurtosis: 1.20 to 1.40). The Pearson correlation coefficient between the two administrations was *r* = 0.48 (*p* = 0.001), indicating a moderate, positive, and statistically significant relationship. These findings suggest that the scale demonstrates acceptable stability over time.

## Discussion

This study contibutes to nursing education by presenting empirical psychometric evidence for a newly developed scale that evaluates nursing students’ knowledge, attitudes, and practices related to evidence-based care bundle implementation. The results indicate that the Care Bundle Practice, Knowledge, and Attitude Scale demonstrates adequate content and construct validity, as well as acceptable reliability, confirming its appropriateness as a psychometrically robust measurement instrument.

### Content validity

In this study, content validity was established by obtaining the opinions of six experts with academic and clinical experience in care bundle implementation. Based on the expert evaluations, the items were found to adequately represent the intended construct in terms of scope, clarity, and relevance. The number of experts consulted falls within the range recommended in the literature, supporting the methodological rigor of the content validity assessment. The fact that the items addressing the knowledge, attitude, and practice dimensions of care bundle implementation were deemed appropriate by the experts indicates that the conceptual framework of the scale was constructed in alignment with the existing literature. This finding suggests that the developed scale has sufficient coverage of the core components of care bundle implementation and a strong capacity to represent the targeted construct. Consistent with this approach, previous scale development studies have reported that content validity assessments based on a limited but field-specific panel of experts are both sufficient and acceptable for establishing content validity [24, 37–41].

### Construct validity

Prior to factor analysis, the suitability of the data for construct validity testing was evaluated using the Kaiser–Meyer–Olkin (KMO) measure and Bartlett’s Test of Sphericity. In this study, a KMO value of 0.88 and a statistically significant Bartlett’s test result (*p* < 0.001) indicate that the sample size was highly adequate for factor analysis and that the relationships among items were based on a strong structural foundation [27, 36, 42]. These findings suggest that the developed scale is not built on random or weak associations but rather reflects a statistically coherent construct. Moreover, this strong factorable structure indicates that the scale has the potential to produce stable and consistent results when applied to different samples in future studies, thereby providing users with a reliable measurement tool.

The results of the exploratory factor analysis demonstrated that the items clustered under three distinct subdimensions, reflecting a meaningful conceptual organization of the construct related to care bundle implementation. According to the literature, factor loadings of at least 0.30 are considered acceptable, while values of 0.60 or higher indicate strong structural contribution [29, 42]. In the present study, Items 11 and 16, which did not meet the established criteria, were removed to strengthen the structural integrity of the scale. This decision enhanced the clarity of the factor structure by ensuring that each retained item more clearly represented its corresponding subdimension and by minimizing potential measurement error caused by weak or overlapping items. As a result, the final version of the scale, consisting of 15 items, offers users a clearer, more consistent, and more interpretable measurement structure.

The accuracy and stability of the three-factor structure identified through exploratory factor analysis were subsequently tested using confirmatory factor analysis [25]. The acceptable fit indices obtained from the confirmatory factor analysis indicate that the scale statistically supports the theoretically anticipated dimensions of knowledge, attitude, and practice related to care bundle implementation. This confirmation demonstrates that the scale not only meets statistical requirements but also successfully reflects the conceptual framework underlying care bundle competencies. The validation of this three-dimensional structure suggests that the scale can generate construct-valid and meaningful results in both educational evaluations and research applications.

### Reliability analysis

Reliability refers to the degree to which a measurement instrument produces consistent and stable results over time. For a scale to be considered reliable, it is expected to yield similar results when administered repeatedly under comparable conditions.

In this study, reliability was evaluated in terms of both internal consistency and temporal stability. The overall Cronbach’s alpha coefficient of the scale was found to be 0.83, indicating a high level of internal consistency for the measurement tool as a whole. The alpha coefficients calculated for the subdimensions were 0.83 for Practice, 0.62 for Attitude, and 0.66 for Knowledge, demonstrating that each subdimension exhibits an acceptable level of internal consistency. These findings indicate that the scale is capable of assessing the knowledge, attitude, and practice components of care bundle implementation in a consistent yet distinct manner. From an educational and research perspective, the ability to interpret subdimension scores separately allows users to more clearly identify specific areas in which students may require further development [32].

The temporal stability of the scale was assessed using the test–retest method. The close similarity between the mean scores obtained from the first and second administrations conducted four weeks apart (58.44 ± 7.81 and 57.74 ± 8.36, respectively) indicates that the scale maintains measurement stability over time. This finding suggests that the developed instrument is not sensitive to short-term fluctuations and yields stable results across repeated measurements. Such stability is particularly important for educational evaluations and research designs involving follow-up assessments or pre- and post-intervention comparisons, supporting the use of the scale in longitudinal applications [27, 32, 42].

Taken together, these findings demonstrate that the Care Bundle Practice, Knowledge, and Attitude Scale produces consistent measurements and exhibits adequate internal consistency and temporal reliability. The ability of the scale to generate reliable results at both the total score and subdimension levels supports its use as a dependable measurement instrument in nursing education, educational assessment, and research contexts.

## Conclusion and recommendations

In this study, a psychometrically sound measurement tool was developed to assess nursing students’ knowledge, attitudes, and practices related to evidence-based care bundle implementation, addressing a well-documented gap in the nursing education literature. To the best of the authors’ knowledge, this study is among the first to focus explicitly on the systematic assessment of educational competencies required for care bundle implementation. While existing research on care bundles has largely concentrated on their effectiveness in improving clinical outcomes, relatively limited attention has been given to measuring the educational readiness necessary for their successful application. By shifting the focus toward knowledge, attitudes, and practices within an educational framework, this study contributes to the nursing education and evidence-based practice literature by enabling a more structured and systematic evaluation of students’ preparedness for care bundle implementation.

The findings indicate that the Care Bundle Practice, Knowledge, and Attitude Scale provides a theoretically grounded and methodologically framework for assessing key dimensions of care bundle competency among nursing students. By facilitating the identification of learning needs and the monitoring of competency development, this scale has the potential to inform curriculum planning and guide the evaluation of targeted educational interventions aimed at strengthening evidence-based nursing education.

Beyond its educational implications, the use of a standardized and validated assessment tool may also indirectly contribute to improvements in the quality of patient care by supporting the development of nurses who are better prepared to implement care bundles consistently and effectively in clinical settings. However, as the scale was validated within a student popuation, its use should be interpreted within this context. Future research is therefore recommended **to** extend the validation of this instrument to practicing nurses, diverse clinical **settings**, different cultural contexts, in order to enhance its generalizability and support its broader application in both educational and clinical practice settings.

### Implications for nursing education and practice

The availability of a validated and psychometrically robust instrument for assessing nursing students’ competencies in care bundle implementation represents a significant contribution to nursing education. The Care Bundle Practice, Knowledge, and Attitude Scale can be utilized to systematically evaluate learning outcomes associated with evidence-based practice, identify specific areas in which students may require additional educational support, and **inform** curriculum development and instructional planning, as well as the design of targeted educational interventions. Further researches are recommended to examine the applicability and performance of this scale across diverse educational and clinical contexts, including samples of practicing nurses, in order to enhance its generalizability and broaden its contribution to evidence-based nursing education and practice.

### Limitations of the study

An important limitation of this research is that the scale was developed and validated **exclusively** within a sample of nursing students. Consequently, the findings may have limited applicability to practicing nurses, as their professional responsibilities and clinical experiences may differ substantially from those of students. Future studies should therefore validate the scale among nurses working in various healthcare settings to further establish its reliability and construct validity. In addition, the study sample consisted of third- and fourth-year nursing students from a single institution, which may further restrict the generalizability of the findings.

Although test–retest reliability analysis demonstrated temporal stability over a four-week interval, the long-term stability of the scale could not be evaluated. Accordingly, longitudinal studies with longer follow-up periods are warranted to determine whether the measurement properties of the instrument remain stable over time and to assess its sensitivity to educational or clinical interventions.

Furthermore, while obtaining opinions from six experts for content validity is considered methodologically acceptable, validation with a larger and more diverse panel of experts may further strengthen the content validity of the scale. Future research should also include larger samples with diverse socio-cultural characteristics and conduct cross-cultural adaptation and validation studies to enhance the generalizability and broader applicability of the scale in both nursing education and clinical practice settings.

## Supplementary Information

Below is the link to the electronic supplementary material.

Supplementary Material 1

## Data Availability

The datasets used and/or analyzed during the current study are available from the corresponding author upon reasonable request.
