# Supplementary material for: Development and psychometric evaluation of a scale assessing nursing students’ knowledge, attitudes, and practices regarding evidence-based care bundles
Source: BMC Nurs. 2026 Feb 28;25:316. doi: 10.1186/s12912-026-04488-0 (PMC13059174; doi:10.1186/s12912-026-04488-0)
Supplement: Supplementary file 1 — Supplementary Material 1 [file 12912_2026_4488_MOESM1_ESM.docx]

| \| **A SCALE DEVELOPMENT STUDY: CARE PACKAGE PRACTICE, KNOWLEDGE AND ATTITUDE SCALE** \| \| --- \|   **Dear Participant** This study has been designed to evaluate the awareness of **care package practices** that improve the quality of care in clinical settings, focusing on knowledge, attitudes, and practices. The results of this survey will be used in the research entitled **“A Scale Development Study: Care Package Practice, Knowledge and Attitude Scale.”** Your sincere and accurate responses to the questions in the survey will be used solely for academic purposes, and all information provided will be kept strictly confidential. Your contribution to this study is of great importance. We sincerely thank you in advance for your interest and the time you have devoted.  **Öğr. Gör. Dr. Tuğba DEMİROĞLUDOST Öğr. Gör. Dr. Gülistan YURDAGÜL** |
| --- | --- |

| **Student Information Form**  **No:** | |
| --- | --- |
| Age |  |
| Gender | Female ( ) Male ( ) |
| Marital Status | Married ( ) Single ( ) |
| Educational Status | Undergraduate (Ongoing) |
| Current Year of Study | 1 ( ) 2 ( ) 3 ( ) 4 ( ) |
| Have you previously received training related to care packages? | Yes ( ) No ( ) |
| Are care packages implemented in the clinics where you have had practice? | Yes ( ) No ( ) |

| No | Nurses play a primary role in the implementation of care packages. For this reason, the opinions of nursing students are important. You are asked to read each statement carefully and rate yourself. The response options are: Strongly Disagree (1), Disagree (2), Neither Agree nor Disagree (3), Agree (4), Strongly Agree (5). Please mark the degree that best reflects how you feel and do not leave any item blank. | Strongly Disagree | Disagree | Neither Agree nor Disagree | Agree | Strongly Agree |
| --- | --- | --- | --- | --- | --- | --- |
| 1 | The care package implementation aims to provide the highest level of care. |  |  |  |  |  |
| 2 | A care package is a set of methods used to standardize care and treatment in hospitals. |  |  |  |  |  |
| 3 | A care package is not a checklist, but rather a personalized care plan. |  |  |  |  |  |
| 4 | The care package is developed based on scientific evidence. |  |  |  |  |  |
| 5 | Interventions in the care package are designed to be applicable to every patient. |  |  |  |  |  |
| 6 | Care packages are prepared according to the needs of the institution. |  |  |  |  |  |
| 7 | A care package is prepared not for the patient personally, but for the disease, symptom, or medical condition. |  |  |  |  |  |
| 8 | The care package is implemented by a multidisciplinary team. |  |  |  |  |  |
| 9 | Care package applications should be used in a defined patient population in a specific setting. |  |  |  |  |  |
| 10 | Care package applications include at least 3 and at most 5 interventions whose effectiveness has been proven. |  |  |  |  |  |
| 11 | Each intervention in the care package is partially independent of the others. |  |  |  |  |  |
| 12 | Interventions in the care package are applied as a whole, following the all-or-none principle. |  |  |  |  |  |
| 13 | Interventions in the care package are applied simultaneously. |  |  |  |  |  |
| 14 | For a care package to be successful, all interventions within it are applied sequentially as a whole. |  |  |  |  |  |
| 15 | Feedback is provided to implementers during care package application. |  |  |  |  |  |
| 16 | The care package implementation increases the workload in clinics. |  |  |  |  |  |
| 17 | The care package implementation improves the quality of healthcare services. |  |  |  |  |  |
